# Small Molecule Identification with MOLGEN and Mass Spectrometry

**DOI:** 10.3390/metabo3020440

**Published:** 2013-05-28

**Authors:** Markus Meringer, Emma L. Schymanski

**Affiliations:** 1 DLR: German Aerospace Center, Earth Observation Center (EOC), Münchner Strasse 20, D-82234 Oberpfaffenhofen–Wessling, Germany; E-Mail: markus.meringer@dlr.de; 2 Eawag: Swiss Federal Institute of Aquatic Science and Technology, Überlandstrasse 133, CH-8600 Dübendorf, Switzerland

**Keywords:** CASMI, mass spectrometry, MOLGEN, MOLGEN–MS, MOLGEN–MS/MS, small molecule identification, structure generation, structure elucidation

## Abstract

This paper details the MOLGEN entries for the 2012 CASMI contest for small molecule identification to demonstrate structure elucidation using structure generation approaches. Different MOLGEN programs were used for different categories, including MOLGEN–MS/MS for Category 1, MOLGEN 3.5 and 5.0 for Category 2 and MOLGEN–MS for Categories 3 and 4. A greater focus is given to Categories 1 and 2, as most CASMI participants entered these categories. The settings used and the reasons behind them are described in detail, while various evaluations are used to put these results into perspective. As one author was also an organiser of CASMI, these submissions were not part of the official CASMI competition, but this paper provides an insight into how unknown identification could be performed using structure generation approaches. The approaches are semi-automated (category dependent) and benefit greatly from user experience. Thus, the results presented and discussed here may be better than those an inexperienced user could obtain with MOLGEN programs.

## 1. Introduction

Mass spectrometry generally provides quite comprehensive information about the identity of an unknown compound, even at very low concentrations and is thus highly sensitive and also selective. The mass to charge ratio (*m*/*z*) of the molecular ion, along with isotope patterns and fragment peaks, helps to identify the molecular mass of the analyte and thus the molecular formula. The fragmentation pattern also gives insight into the presence or absence of substructures in the molecule and thus can guide the way to the correct structural formula. However, none of these steps are trivial and multiple solutions typically appear valid. For instance, multiple molecular formulas have the same molecular mass and multiple structural formulas have the same molecular formula and even the same substructures. Dedicated instrumentation and sophisticated algorithms are necessary to collate the information available in order to identify an unknown compound.

The Critical Assessment of Small Molecule Identification (CASMI) contest was initiated in 2012 to enable the comparison of different experimental and computational techniques for small molecule identification on a common set of mass spectrometry data. The four categories of the CASMI contest were suited to different MOLGEN programs.

CASMI Category 1, best molecular formula using high resolution LC–MS/MS data, was ideal for the most recent MOLGEN development, MOLGEN–MS/MS [1]. This command line program accepts the MS and MS/MS data and calculates all molecular formulas matching the restrictions, using the isotope pattern match of the MS data and the number of MS/MS peaks with an assigned subformula to score the molecular formula candidates. The resulting output includes a mass deviation (ppm), the MS match value (MS MV), the MS/MS match value (MS/MS MV) and the combined match value (combMV), a direct multiplication of the MS and MS/MS MVs. Many other options exist for users to control the output, including an existence filter, fuzzy formula and element restriction options, as well as several scoring alternatives.

CASMI Category 2, best (structural) identification using high resolution LC–MS/MS data, was approached using structure generators alone, adding restrictions manually. The versions currently available are MOLGEN 3.5 [2,3] and the newer development, MOLGEN 5.0 [4,5]. Both of these generate structures that match the molecular formula(s) and optional structural restrictions provided by the user, but are implemented differently. MOLGEN 3.5 allows the incorporation of substructure information using macroatoms and “good list” structures to define substructures that are present in the candidates, with “bad list” structures used to exclude certain substructures. Although good list and bad list items may overlap, macroatoms should not overlap each other as these are effectively “building blocks” of the molecule. The definition of macroatoms allows for more efficient generation. In contrast, MOLGEN 5.0 uses a system of “prescribed” and “forbidden” structures, without the definition of macroatoms, but has additional functionality such as definition of atom type restrictions. As no fully-built computer-aided structure elucidation (CASE) system was available for CASMI Category 2, the ranking of candidates was performed using external programs. Of the openly accessible *in silico* fragmenters, MetFrag (see [6]) was better suited to many candidates than the more computationally intense FiD (see [7]). Both use the bond-disconnection approach, as opposed to the rule-based approach of Mass Frontier [8] and MOLGEN–MS (see below). MOLGEN–QSPR, which is capable of calculating many different molecular properties [9], was used to generate steric energy values for candidate ranking.

CASMI Categories 3 and 4 were suitable for MOLGEN–MS [10], the *de novo* structure elucidation system for low resolution electron impact mass spectrometry (EI–MS), usually coupled with gas chromatography (GC–MS). For more details, see, e.g., [11,12]. The three classical steps of an automated structure elucidation system proposed in the DENDRAL project [13] (plan–generate–test) are implemented in MOLGEN–MS. In the first step (plan), structural properties are derived from the spectral data using the module *MSclass*, an integrated implementation of the mass spectral classification software by Varmuza and Werther [14]. In the second step (generate), structures fulfilling the properties from the planning step are generated using the MOLGEN 4.0 [15] kernel. In the third step (test), the generated structures are fragmented *in silico* according to standard mass spectrometric fragmentation rules and the resulting fragments are compared with the fragments in the experimental spectrum [16]. Ideally, the correct structure should be the best match, but this is rarely the case especially with many candidates. Prior to structure generation with MOLGEN–MS, a similar approach is used to derive the molecular formula. *MSclass* results provide information about the absence, presence and multiplicities of certain elements. These can be used as input for the molecular formula generator to reduce the number of possible formulas. Generated formulas are then tested against isotope pattern of the molecular and fragment ion peaks in the spectrum. Two modules for molecular formula calculation are available in MOLGEN–MS. The first module, *MolForm*, fits the molecular formula using the isotope pattern and the restrictions provided. The second module is named *ElCoCo* (*El*emental *Co*mposition *Co*mputation) and uses the whole spectrum, which provides further information in the case that *MolForm* is not sufficient. Algorithmic details are described in [11,17,18].

While MOLGEN–MS was built to be a stand-alone, database-independent spectral interpretation interface, the results in [16,19] showed that MOLGEN–MS alone was insufficient for routine structure elucidation. However, enhancing MOLGEN–MS with additional information obtained from the NIST database [20] and calculated properties (where available) greatly increased the chances of successful structure elucidation [19,21]. More recently, the ‘consensus scoring’ approach [22] heralded a change in strategy away from structure ‘filtering’ towards an integrated scoring approach. Structures that satisfied more additional criteria (or properties) with higher match values achieved higher scores than those with lower spectral match values or matching fewer of the additional criteria. These additional criteria included partitioning behaviour, retention behaviour and also steric energy. While many different programs were considered in [22], we restricted the calculations for CASMI to MOLGEN software where possible.

The aim of this article is to demonstrate structure elucidation using the different MOLGEN programs on the CASMI challenges. Thus, the challenges as well as the results of the other participants in the CASMI contest are described as far as necessary, but detailed descriptions were beyond the scope of this article. More extensive details on the challenges can be found in [23], while details about the participants and their results are reviewed in [24].

## 2. Methods

This section includes the parameters and reasoning used for the CASMI challenges and introduces measures to evaluate the results presented in this article.

### 2.1. Category 1: Best Molecular Formula with LC–MS/MS

MOLGEN–MS/MS [25] was used to calculate the entries for Category 1. The elements were restricted to C, H, N, O, P and S, as there was no evidence of halogens in any challenge (“el = CHNOPS”). The existence filter (“exist”, which restricts the results to molecular formulas where at least one structural formula is possible) was used, while both odd and even electron ions were allowed to explain MS/MS fragments (“oei”). As all data was quoted to be below 5 ppm accuracy, 5 ppm was used for MS matching (“ppm = 5”) and 10 ppm for MS/MS matching (“acc = 10”) since research shows that these are appropriate settings for Orbitrap MS/MS data [26]. For more details on the parameters see [1,25].

The information provided by the organisers and summarised in the listing below was used to decide whether calculations were performed with positive or negative ionisation. Where multiple MS/MS files were available, they were combined into one file with all peaks present, taking the peak with the highest intensity where multiple peaks were present within 10 ppm.

The exact parameters used were as follows:

– Challenges 1, 4–6, 10, 12–15, 17: el = CHNOPS, ppm = 5, acc = 10, oei, exist, ion = +H.
– Challenges 2–3: el = CHNOPS, ppm = 5, acc = 10, oei, exist, ion = −H.
– Challenge 11: el = CHNOPS, ppm = 5, acc = 10, oei, exist, m = 232.088, ion = −e. Here, m = 232.088 set the mass for M^+^, and ion mode ‘−e’ corresponds with M^+^.
– Challenge 16: el = CHNOPS, ppm = 5, acc = 10, oei, exist, m = 359.1481 ion = +e. Here, m = 359.1481 was used to set an M^+^ ion mass, with ion mode +e to obtain the corresponding formulas.

The combined MS and MS/MS MV (combMV) was used to score the candidates, except for Challenge 16, where only the MS/MS MV was used because the MS MV was zero.

The release of the preliminary evaluation in early February 2013 revealed that the parameter chosen for the MS/MS accuracy for Challenges 1–6 was incorrect and that the MS data for Challenges 2, 4 and 6 were unexpectedly outside the 5 ppm error margin given by the organisers. As a result, new entries were submitted for these challenges, using recalibrated data provided only after the close of the competition by the organisers for Challenges 2, 4 and 6 and the original files for Challenges 1, 3 and 5 with 5 ppm error for the MS and 60 ppm error for the MS/MS. All other parameters were left as above. The MS/MS parameter was revised using the correct answer to optimise the parameter selection, as the solutions were released with the preliminary evaluation. The value of 60 ppm was chosen based on the subformula assignment results of MOLGEN-MS/MS, as most “true” MS/MS peaks could be assigned a formula with this error margin, while using a smaller ppm error resulted in some true MS/MS peaks without a formula assigned. For higher accuracy data (5–20 ppm) this parameter selection can also be performed without knowledge of the solution, by investigating the error at which no additional peaks can achieve a subformula for any of the molecular formulas possible.

### 2.2. Category 2: Best Structure with LC–MS/MS

The submissions for this category required significant manual intervention as there is currently no integrated “MOLGEN” solution for high accuracy MS/MS data. The classifier interpretation and virtual fragmentation systems in MOLGEN–MS cannot be applied directly to high accuracy data without modification since different ionisation techniques and fragmentation pathways are relevant. An expansion to account for accurate mass binning, not unit mass, would also be necessary. Thus, the spectral interpretation was performed manually using prior knowledge and an alternative *in silico* fragmenter, MetFrag, was used to rank the candidates. Since manual interpretation is a time-consuming process and no specific classifiers for LC-MS/MS spectra exist (as far as the authors are aware), entries were only attempted for challenges where it was considered that the molecular formula and substructure information were (reasonably) certain. Substructure information was obtained from the MOLGEN–MS/MS output (fragments or losses associated with common groups) or from spectral interpretation based on previous experience. In the end, submissions were made for 6 of the 14 challenges where the correct formula was clear and sufficient substructure information was available. Following the release of the evaluation results it was clear that an error had been made in the substructure restrictions for Challenge 17; this was resubmitted following the close of the competition with correct substructures (see Section 3.2 for more details).

Structure generation was performed with MOLGEN 3.5 or MOLGEN 5, with information from the spectral interpretation added as substructures. For MOLGEN 3.5 this information was added as a macroatom or “good list” substructure for substructures considered to be present and as a “bad list” substructure for those suspected to be absent. These substructures were drawn and saved using MOLED [3]. The nitrogen valence was left at the default value of 3 as no evidence of a nitro group was found in the challenges we submitted (these generally give distinct fragmentation patterns). For MOLGEN 5.0 combinations of prescribed and forbidden substructures were used, which were provided as MDL MOL files [27] (other formats are available). Additional features of MOLGEN 5.0 were also used, including the definition of atom states; further details are given in Section 3.2. For all entries, steric energy values were calculated with MOLGEN-QSPR [9]. “M END” lines were added to MOLGEN 3.5 SDF files to avoid compatibility issues. SMILES notation was generated using OpenBabel [28], which was also used to generate SDFs without explicitly-defined aromaticity for Challenge 14. *In silico* fragmentation was performed using MetFrag [6] with the ionisation settings adjusted according to the information on the CASMI website and from the results of Category 1, with mzabs = 0.001 and mzppm = 10. The results of MetFrag and MOLGEN–QSPR were combined into a “consensus score” given below:

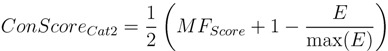

where *E* represents the steric energy and *MF_Score_* the MetFrag score. Note for Challenge 17 the command line version of MetFrag was used due to the large number of candidate structures: this version has a slightly different scoring scheme to the web interface.

### 2.3. Category 3: Best Molecular Formula with GC–MS

MOLGEN–MS [10,29] was used to calculate the entries for Category 3, with additional information from the NIST database included manually. For each challenge, the MSP file from the CASMI website [30] was sent to a NIST library search [20] to obtain substructure information. Following this, the CSV file for each challenge was imported into MOLGEN–MS and the *MSclass* module was run to obtain the database-independent substructure classifiers complementary to the NIST information. The information from NIST and *MSclass* was then used to formulate restrictions for the first molecular formula calculation with the *MolForm* module. The exact information used for the individual challenges is presented below. In *MolForm*, the formulas are scored according to the deviation between the experimental isotope distribution measured for the M^+·^ ion and the theoretical distribution calculated for each matching formula. As the smallest deviation represents the closest match, this was adjusted to match the CASMI scoring requirements [31] according to the following:

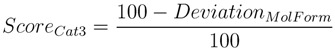

resulting in a score between 0 and 1, where 1 is the best match and 0 the worst.

For two challenges, 11 and 12, the M^+^^·^ ion was not present and the calculation with *MolForm* was not possible. In these cases the *ElCoCo* module was used, which uses the full spectrum to match the formulas, not just the isotope pattern of the M^+^^·^ ions. Again, the full information is given below. The *ElCoCo* formulas are given a score between 0% and 100%, the higher the better. These scores were divided by 100 to make them consistent with the score above, although the values are not comparable.

The outputs of *MolForm* and *ElCoCo* were saved to a text file through the MOLGEN–MS interface and imported into Excel, where the scoring conversions mentioned above were performed.

The restrictions used, challenge-by-challenge, were as follows (NIST and MOLGEN–MS information combined for simplicity):

– Challenge 1: C ≥ 7, O ≥ 2, H unlimited, F, Cl, Br, I, N, P, S, Si = 0. RDB = 6–7.
– Challenge 2: C ≥ 7, O ≥ 2, N ≥1, H unlimited, F, Cl, Br, I, P, S, Si = 0. RDB = 7–8.
– Challenge 3: O, Cl = 1, C, H unlimited. F, Br, I, N, P, S, Si = 0. RDB = 4.
– Challenge 4: O, Cl = 1, C, H unlimited. F, Br, I, N, P, S, Si = 0. RDB = 4.
– Challenge 5: Cl = 2, C, H unlimited. F, Br, I, N, P, S, O, Si = 0. RDB = 4.
– Challenge 6: C, H unlimited. F, Cl, Br, I, N, P, S, O, Si = 0. RDB = 8. MW = 154. Note: the two highest peaks in the CSV file were removed to improve results; likely impurities.
– Challenge 7: O = 1, Cl = 2, C, H unlimited. F, Br, I, N, P, S, Si = 0. RDB = 5.
– Challenge 8: C, H unlimited. F, Cl, Br, I, N, P, S, O, Si = 0. RDB = 9.
– Challenge 9: O = 1–2, Cl = 1, C, H unlimited. F, Br, I, N, P, S, Si = 0. RDB = 5.
– Challenge 10: O = 1, Cl = 3, C, H unlimited. F, Br, I, N, P, S, Si = 0. RDB = 4.
– Challenge 11: MW = 257; N = 1, O, S = 2–20, P = 1–20, C, H ≥ 4. F, Br, I, Si = 0.
– Challenge 12: MW = 288; C ≥ 6, H ≥ 1, Cl ≥ 5. F, Br, I, N, P, S, O, Si = 0. RDB = 1.
– Challenge 13: S = 1–20, C, H unlimited. F, Cl, Br, I, N, P, S, Si = 0.
– Challenge 14: C ≥ 1, H ≥ 3, O= 3, P, S ≥ 1. F, Cl, Br, I, N, Si = 0.
– Challenge 15: C ≥ 12, H, N ≥ 0, O ≥ 1. F, Cl, Br, I, P, S, Si = 0. RDB = 9.
– Challenge 16: C, P, S ≥ 1, H ≥ 3, O = 2, N unlimited. F, Cl, Br, I, Si = 0.

Note: The RDB values (Ring and Double Bond count) were not always used explicitly to restrict the candidates in the entries for Category 3; however they were used to select the formula before moving on to Category 4.

### 2.4. Category 4: Best Structure with GC–MS

Similarly to Category 3, MOLGEN–MS [10,29] was used to calculate the entries for Category 4, with additional information from the NIST database included manually. The formula calculated as part of Category 3 was used for input into the *MolIn* module, along with the MOLGEN–MS and NIST classifier information. The classifiers were checked for consistency with the molecular formula. Moving onto the *MOLGEN* module, all structures were generated fitting the given restrictions. The substructures used are given in Figures A1–A15 in the appendix.

Following structure generation, all structures were fragmented in the MOLGEN–MS module *ReNeGe* (*Re*action *Ne*twork *Ge*nerator) to generate the MOLGEN–MS match value for ranking the candidate structures. Following the ranking, the structures (including the match value) were exported as SDF for further processing.

For Challenges 1 and 2, standards were present for the calculation of retention indexes but were not used due to the detailed substructure information available. As such, the only additional information used for candidate selection was the steric energy, calculated with MOLGEN-QSPR [9]. The steric energy was calculated by importing the SDF from MOLGEN–MS, adding hydrogens, calculating the 3D layout with 10 iterations and finally calculating the steric energy index. The resulting value (kcal/mol) was exported as a text file with the structure number. For Challenges 1 and 2, the resulting “consensus score” was

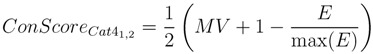

where *E* represents the steric energy and *MV* the MOLGEN–MS match value.

For Challenges 3–16, partitioning information (log *K_ow_*) was given and was incorporated into the candidate selection for these challenges. The log *K_ow_* values were calculated with the EPI Suite*^TM^* *Kowwin* module in batch mode. If the candidate structure had an estimated log *K_ow_* within the given range ± 1, this was considered a match (*K*_*ow*0,1_ = 1); if the log *K_ow_* was outside this range, it was not considered a match (*K*_*ow*0,1_ = 0). The steric energy was also considered for these challenges as for Challenges 1 and 2. The resulting consensus score for Challenges 3–16 for Category 4 was:

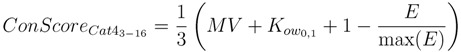



### 2.5. Evaluation Measures and Ranking

Two measures, in addition to the absolute rank, were used to assess the results presented in this article. One of these is the relative ranking position, RRP, which is defined here as

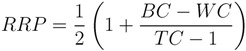

where BC, WC and TC are the better, worse and total candidates, respectively. As opposed to the RRP used in CASMI [24], RRP = 0 is best, RRP = 1 is worst and the values are comparable with previous calculations (RRP = 0.273 for MOLGEN-MS, see e.g., [32]). Another measure is the number of possible structures for a given formula. These were generated using MOLGEN 3.5, with atom valences consistent with those used for the CASMI challenges. Generation was restricted to 100,000,000 structures (indicated by >1E8 in the tables when this limit was reached) and an estimate of the percent of all possible structures covered (from MOLGEN 3.5) is provided for these cases.

## 3. Results and Discussion

### 3.1. Category 1: Best Molecular Formula with LC–MS/MS

The results for this category are presented in Table 1.

**Table 1:** MOLGEN–MS/MS results for Category 1 (best molecular formula with LC–MS/MS data). Numbers in brackets denote the number of candidates with equal scores, ranks shown are best case absolute ranks. NA is not applicable (*i.e*., no results). Remaining abbreviations: see text.

Challenge Number	Molecular Formula	PubChem CID	Formulas Entered	Rank with MS MV	Rank with MS/MS MV	Rank with combMV	Rank of Winner
1	C_18_H_36_N_4_O_11_	6032	54	12	1 (38)	11	1
2	C_28_H_32_O_14_	5280665	249	1	1 (239)	1	1
3	C_14_H_27_NO_9_S_3_	46173875	90	1	27 (25)	14	1
4	C_19_H_17_NO_4_	12304178	15	1	1 (10)	1	1
5	C_19_H_23_NO_4_	10233	10	1	1 (13)	1	1
6	C_21_H_21_NO_6_	197775	31	1	1 (19)	1	2
10	C_14_H_9_NO_2_	6710	3	1	1	1	1
11	C_17_H_12_O	104977	6	1 (6)	1 (4)	1 (4)	NA
12	C_17_H_16_N_4_O_4_	221491	18	1 (18)	1 (4)	1 (4)	NA
13	C_19_H_17_OP	76293	10	1	1 (3)	1	1
14	C_12_H_9_N	98617	2	1	1	1	1
15	C_12_H_13_NO_2_	2145522	2	1	1 (2)	1	1
16	C_18_H_21_N_3_O_5 _	18091616	20	NA	2 (3)	2 (3)	NA
17	C_13_H_13_N_3_	68380	3	1	1	1	1

Nine of the 14 entries were “unique” number 1 ranks, specifically Challenges 2, 4–6, 10, 13–15 and 17, while two of the remaining five entries had the correct formula in equal top place with three other formulas (Challenges 11 and 12). These are shown as rank 1 in Table 1, although this was assessed at an absolute rank of 4 in the evaluation performed by the CASMI organisers [24]. For these two challenges, the MS MVs were the same for all candidates due to the non-standard ionisation behaviour and thus all formulas with the same number of fragments matching a subformula had the same score. Not even a more sophisticated weighting of the mass or intensity of the fragments would have helped here as the same peaks were explained for all equally-ranked formulas. The error margin of 60 ppm for the MS/MS was very large and resulted in multiple subformulas for many of the peaks in the earlier challenges, as is apparent in the number of candidates with equal MS/MS MV scores in Table 1.

Only three challenges did not have the correct formula scored the highest: Challenges 1, 3 and 16. Challenge 1 showed a distinct improvement in the ranking with the more appropriate error margins of 60 ppm, with the correct formula ranked 11*^th^* and not 23*^rd^* in the earlier submission. This improvement is due to the increased subformula assignment for the correct formula. This was the only challenge where the MS MV was not highest for the correct formula; greater accuracy in the MS/MS may have improved this rank further. Challenge 3 was the worst result and the only case where using the combMV worsened the rank of the correct molecule, most likely due to the large errors in the MS/MS. Challenge 16 underwent in-source fragmentation and thus the MS MV was 0 as neither parent ion nor isotope peaks were present. Three formulas, including the correct one, explained the same number of MS/MS peaks, but one incorrect formula explained some additional peaks, improving its ranking above the correct formula. In reality, it would have been very difficult to detect the in-source fragmentation and deduce the correct parent mass; only the fact that the neutral mass of the molecule was given by the organisers enabled the submission of the correct answer. Interestingly, Table 1 shows that the MS MV determined the rank in most cases, although this is not always true (see e.g., [1]).

In the end, eight of the 14 submissions were ranked equally with the CASMI winner, one exceeded the rank of the CASMI winner (Challenge 6, although with the corrected data, unlike the CASMI winner) and results were obtained for three challenges where no winner was declared (*i.e*., no external participant obtained the correct answer).

### 3.2. Category 2: Best structure with LC–MS/MS

As this category did not have an integrated MOLGEN solution, submissions were only made for six of the 14 challenges with informative MS and MS/MS spectra. These results are shown in Table 2.

Table 2 clearly shows the reduction in structure numbers from >100,000,000 to below 1500 in all 6 cases and even below 50 in 4 cases. This highlights the critical role that the substructure information plays in identification using structure generation approaches. However, the restrictions for candidate generation were provided manually based on experience. As these challenges were provided by one of the authors (ES), the selection of substructure restrictions was made significantly easier (and consequently also not truly unbiased) due to extensive prior experience with these and similar compounds. Thus, these results may be close to a ‘best case scenario’ for these challenges. Since Challenges 10–17 are relatively small molecules, they are also well suited to structure generation approaches. The lack of experience with mass spectrometry of natural products and plant metabolites as well as the lack of certainty about the correct molecular formula (see the previous section) contributed to the decision not to enter Challenges 1–6. CASMI winners were declared for three of the six challenges in Table 2 and the MOLGEN ranks were surprisingly comparable, especially considering that the winner of Challenges 13 and 15 used spectral libraries [24,33].

**Table 2:** MOLGEN results for Category 2 (best molecular structure for LC–MS/MS). NA is not applicable (*i.e*. no results).

Challenge Number	Molecular Formula	PubChem CID	Structures Possible(%)	Structures Entered	MOLGEN Rank	MOLGEN RRP	Rank of Winner
10	C_14_H_9_NO_2_	6710	>1E8 (<1%)	171	63	0.365	NA
11	C_17_H_12_O	104977	>1E8 (<1%)	8	3	0.286	NA
13	C_19_H_17_OP	76293	>1E8 (<1%)	4	3	0.667	1
14	C_12_H_9_N	98617	>1E8 (18%)	41	22	0.525	12
15	C_12_H_13_NO_2_	2145522	>1E8 (1.5%)	32	26	0.806	1
17	C_13_H_13_N_3_	68380	>1E8 (<1%)	1295	58	0.044	NA

**Figure 1:**
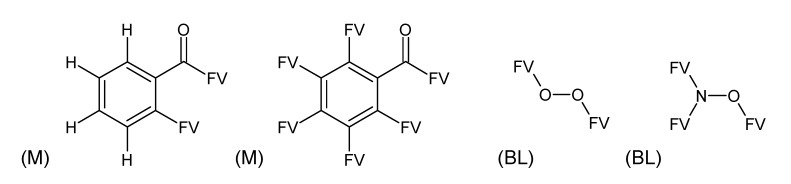
Substructures for Challenge 10. Letters to the bottom left indicate (M) macroatom; (BL) bad list (forbidden) structures. FV = free valence.

*Challenge 10* (C_14_H_9_NO_2_; 1-aminoanthraquinone):

The MS/MS of this compound contained some distinctive fragments and losses. The loss at 105.033 suggested a benzaldehyde substituent, while the losses of water and carbonyl groups combined with the high ring and double bond equivalents (DBE) suggested a stable molecule with carbonyl groups contributing to aromaticity. The lack of NO or NO_2_ losses indicated that a nitro group was unlikely to be present, while the lack of an NH_3_ loss appeared to indicate a ring-bound N. A reasonable number of structures were generated by defining two benzaldehyde groups, with one forced to have only one substituent in the ortho-position to allow formation of the aromatic system. As aromatic bridged substances are rare, meta- and para-substitution on one of the aromatic rings could be ruled out, which cut down the number of possible structures dramatically. The resulting substructure restrictions are shown in Figure 1. The resulting number of structures, 171, is many orders of magnitude lower than the number of possible structures for this formula, while the RRP (0.365) is comparable with the RRPs of MOLGEN–MS and Mass Frontier calculated on datasets with less than 200 molecules (0.352–0.393 [12,32]). As some of the fragments observed for this compound result from rearrangement reactions that were not predicted by the *in silico* fragmentation reactions incorporated in MetFrag (one example is the water loss from a carbonyl group), it is possible that this ranking could be improved in the future, for example by adding an additional *in silico* fragmentation approach to the consensus score *ConScore_Cat2_*.

**Figure 2:**
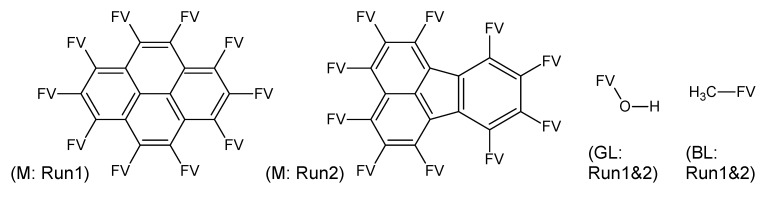
Substructures for Challenge 11. Letters to the bottom left indicate (M) macroatom; (GL) good list (prescribed); (BL) bad list (forbidden) structures. FV = free valence.

*Challenge 11* (C_17_H_12_O; 1-pyrenemethanol):

The MS/MS of this compound contained strong evidence for a stable aromatic compound with only one substituent. The fact that a [M–H]^+^ oxidation product was detected indicated that the OH group was more likely to be present on a substituent, not in the aromatic ring system. No fragments resulted from the aromatic system and it was concluded from the formula that two 4-ring systems were possible. These were provided to MOLGEN as macroatoms (see Figure 2). As there is no “OR” option for macroatoms and defining such groups as good list structures would lead to prohibitively long calculation times, two MOLGEN runs were merged into one for the CASMI submission. The resulting total number of structures is again many orders of magnitude lower than the total number possible for this molecular formula without restrictions. Eight structures were generated: 5 from the fluoranthene skeleton (Run 1) and 3 from the pyrene skeleton (Run 2), as a result of symmetry and aromaticity. Both runs completed in <0.01 s. The final rank, 3 of 8, was almost by chance; without detailed fragments of the aromatic system, spectra of several isomers or retention times, these eight candidates were essentially equivalent for the MS/MS information given. All candidates had the same MetFrag scores and the ranking is purely influenced by the steric energy calculation, which had little relevance as all candidates are equally likely in a chemical sense.

**Figure 3:**
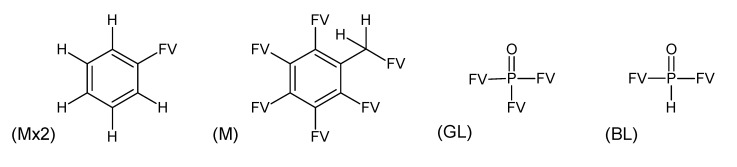
Substructures for Challenge 13. Letters to the bottom left indicate (Mx2) two macroatoms; (M) macroatom; (GL) good list; (BL) bad list structures. FV = free valence.

*Challenge 13* (C_19_H_17_OP; benzyldiphenylphosphine oxide):

The fragmentation patterns arising from this challenge indicated the presence of three aromatic substituents, two phenyl groups and one benzyl or methyl-phenyl group. The remaining part of the formula indicated that it was likely to be a phosphine compound. The corresponding substructure information provided to MOLGEN is shown in Figure 3. Generation was very quick (0.01 s) and resulted in 4 structures after aromatic doublet filtering. Again the final rank of 3 from 4 was almost by chance; all structures had the same MetFrag score so the consensus scoring was influenced purely by very minor differences in steric energy between the structures.

**Figure 4:**
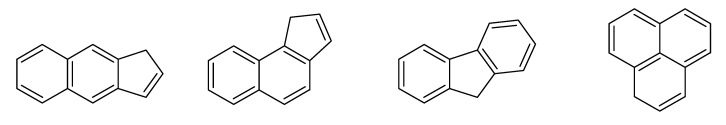
Aromatic skeletons for Challenge 14.

*Challenge 14* (C_12_H_9_N; 1H-benz[g]indole):

Challenge 14 was measured at very high collision energy and all losses seemed to indicate the break-up of an aromatic system. The loss of CHN implied that nitrogen was part of the aromatic system. However, with 13 heavy (*i.e*., non-H) atoms, this aromatic system did not strictly adhere to the Hückel aromaticity rule, which leaves one atom with one H more than usual in an aromatic system. A total of 4 aromatic ‘skeletons’ were possible, shown in Figure 4, which makes defining a macroatom with MOLGEN 3.5 very difficult, since the location of the N or the double bonds cannot be defined. Likewise, defining potential good list structures would have meant effectively hand-drawing all possibilities, which is not the point of a structure generator. Even with only 13 heavy atoms, there are too many molecules possible to generate all molecules using a simpler restriction, e.g., adding a benzene ring only.

MOLGEN 5.0 was the more flexible option in this case, with the ability to define atom types and the functionality to generate the molecules with the required restrictions. The following restrictions were used via the command line options:

– bondsa 11–12: There are 11 or 12 aromatic bonds, corresponding to two condensed aromatic 6–rings (naphthalene skeleton) or two disjoint aromatic 6–rings.
– ringsize 5–13: to avoid rings of size three and four.
– badlist badlist2.sdf: a list of 14 ‘bad’ bridged aromatic substructures, see [4,5].
– badlist SideChainTerminals.sdf: a badlist of substructures that occur at the end of side chains, including –CH_3_, =CH_2_, –NH_2_ and =NH, to prevent the occurrence of side chains.

These restrictions resulted in the generation of 41 structures, approximately eight orders of magnitude reduction from the total number of structures possible with this formula, but with a significantly higher run-time than for MOLGEN 3.5 for similar sized molecules with well-defined macroatoms. For instance, the two runs in Challenge 11 took <0.01 s each; on the same machine MOLGEN 5.0 took exactly 1 h to complete the generation for Challenge 14. However, the resulting 41 structures were much more useful than trying to get a similar output with MOLGEN 3.5 using manually-defined restrictions. Note: a direct run-time comparison cannot be made here as the appropriate macroatoms cannot be defined for this case. The final ranking of 22 of 41 structures was again a reflection of the similarity of all resulting molecules; the top 25 structures had quite high MetFrag scores and the correct structure is the lowest of these. It is likely that spectral information from several isomers would be needed to rank these candidates properly.

**Figure 5:**
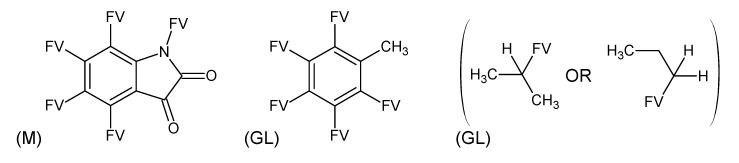
Substructures for Challenge 15. Letters to the bottom left indicate (M) macroatom; (GL) good list; brackets enclose those considered as “or”. FV = free valence.

*Challenge 15* (C_12_H_13_NO_2_; 1-Isopropyl-5-methyl-1H-indole-2,3-dione):

This challenge required a very restrictive macroatom, which almost involved elucidating the full structure by hand. The loss of a C_3_H_6_ group is often an isopropyl substituent, but a propyl substituent could not be ruled out conclusively and thus had to be included in the structure generation. The peak at 91 indicated a methyl-substituted benzene, while the successive loss of water combined with the high DBE and the high collision energies involved in the MS/MS acquisition indicated once again that a stable ring structure with carbonyl groups, not hydroxyl substituents, was likely. The peak at 106 (C_7_H_8_N) indicated that an N was attached to the benzene group, as well as a methyl group. This suggested that the two carbonyl groups must be adjacent and provided enough evidence for the indole-dione macroatom, shown in Figure 5 (along with the other restrictions). The resulting 32 isomers, approximately nine orders of magnitude lower than the total number of structures possible for this formula, were generated within 0.02 s. The ranking is 26 out of 32 and the corresponding RRP = 0.806 is much higher (and thus worse) than the average RRP for ranking using *in silico* fragmentation, even with small structure sets (see above). Although all candidates had relatively high scores, the correct candidate had one of the lowest MetFrag scores (0.839), despite having more peaks explained than other structures. MetFrag cannot explain the water losses resulting from carbonyl groups using the bond-breaking approach, resulting in the lower score. However, by using detailed substructure information and structure generation, the absolute MOLGEN rank of 26 was above the MetFrag result submitted by Ruttkies *et al.* [34], which had an absolute rank of 316 of 2585 possible candidates retrieved by compound database searching. The rank of 26 was, however, not able to compete with the CASMI winner (see Table 2).

**Figure 6:**
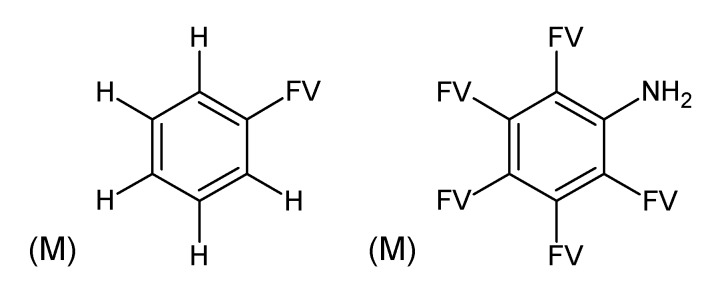
Substructures for Challenge 17. (M) indicates macroatom; FV = free valence.

*Challenge 17* (C_13_H_13_N_3_; Nitrin):

The first CASMI entry for this challenge did not contain the correct solution despite submitting a solution containing 1590 candidates, demonstrating how easy it is to miss the correct structure if an incorrect substructure is added. In this case several fragments containing C_7_H_8_N groups or losses seemed to suggest a methyl substituent, although the lack of a fragment at 91 (C_7_H_7_) should have indicated that this was incorrect. As a chance to resubmit entries was offered once the solutions were out, the correct substructure restrictions were used in a resubmitted entry (which was not improved in hindsight apart from removing the incorrect CH_3_ group). The substructure information used is shown in Figure 6. The presence of the macroatom to the right was justified by the numerous fragments and losses involving a benzene with nitrogen attached, while the NH_2_ group was also the first loss, indicating that not all Ns were bound within a chain or a ring. Although the peak at 77 was very small, it indicated the presence of an unsubstituted phenyl (C_6_H_5_). Being able to define one of the aromatic substituents with fixed Hs instead of free valences was very important to reduce the number of structures and especially bridging structures generated. Despite these restrictions, a total of 1295 structures were generated, which were fragmented using the command line version of MetFrag as this overloaded the web interface. The final rank was 58 of 1295 and the resulting RRP = 0.044 is much lower (and thus better) than the average RRP for MOLGEN-MS of 0.273, indicating an above-average ranking success. The absolute rank was close to the ranks achieved by the other (internal) participants with MetFrag (21) and MetFusion (40) using compound databases, while no external participant submitted the correct answer and thus no CASMI winner was declared for this challenge. The final rank was influenced both by the *in silico* fragmentation and steric energy calculation.

### 3.3. Category 3: Best Molecular Formula with GC–MS

The next two sections contain the results for the GC–MS data, where the entries were calculated using MOLGEN–MS. The results are summarised in Table 3.

**Table 3:** MOLGEN–MS results for Categories 3 and 4 (best molecular formula and structure for GC–MS). NA is not applicable (*i.e*., no results, not defined).

Challenge Number	Molecular Formula	PubChem CID	Formulas Entered	Rank of Corrrect	Possible Structures	Structures Entered	Rank of Correct	RRP
1	C_8_H_4_O_3_	6552	5	1	4,161,969	93	19(2)	0.201
2	C_8_H_5_NO_2_	6550	2	1	38,484,571	80	1	0
3	C_7_H_7_ClO	26799	1	1	62,643	3	3	1
4	C_7_H_7_ClO	12823	1	1	62,643	3	2	0.5
5	C_6_H_4_Cl_2_	13866817	1	1	1323	3	1(2)	0.25
6	C_12_H_10_	6478	2	1	37,720,012	187	5	0.021
7	C_7_H_5_ClO_2_	6079	1	1	507,196	3	1	0
8	C_13_H_10_	6592	1	1	>1E8 (40%)	90	1	0
9	C_8_H_7_ClO_2_	11402	1	1	5,160,746	3	3	1
10	C_6_H_3_Cl_3_O	21106172	1	1	19,969	6	2	0.2
11	C_6_H_12_NO_4_PS_2_	16412	15	1	>1E8 (4%)	45	1	0
12	C_6_H_6_Cl_6_	10468511	1	4	1421	1	1	NA
13	C_3_H_6_S_3_	15959	5	1	102	13	13	1
14	C_3_H_9_O_3_PS	8686	1	1	19,054	1	1	NA
15	C_12_H_8_O	551	1	1	>1E8 (30%)	19	1	0
16	C_3_H_9_PS_2_O_2_	29165	8	1	27,776	1	1	NA

The only challenge with the formula ranked below first place in Category 3 was Challenge 11, which was calculated using *ElCoCo* as the M^+^^·^ ion was not present. Realistically, this challenge was one of those cases where CASE via MS is often unsuccessful because multiple formulas would need to be considered to perform a completely unbiased CASE, which leads to a very high number of candidates and thus a very low chance of success. The remaining challenges were very small molecules and it is not surprising that the formulas were correct with the combined substructure information from NIST and MOLGEN–MS. Since there were no other participants in this category, these results are not discussed in greater detail.

### 3.4. Category 4: Best Structure with GC–MS

**Figure A1:**
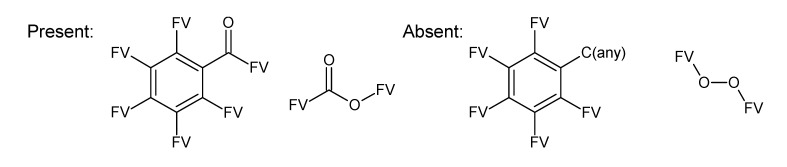
Substructures for GC–MS Challenge 1. FV = free valence.

**Figure A15:**
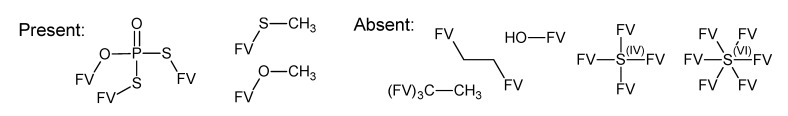
Substructures for GC–MS Challenge 16. FV = free valence.

The results for this category show that CASE via MS, even low resolution MS, is certainly achievable in many cases. Although these molecules are quite small, thousands and even hundreds of thousands of structures are possible for the correct formulas, shown in Table 3. The substructure information from MOLGEN–MS and NIST is essential in limiting the number of candidate structures, by several orders of magnitude in most cases. The detailed substructure information used for these calculations are given in Figures A1–A15 in the appendix.

Of the 16 challenges, eight had the correct structure in first place. This high success rate was mainly due to the low number of candidates generated in many of the challenges because of the successful substructure assignments. Of those eight challenges, three (Challenges 12, 14 and 16) had only one candidate, and the RRP is not defined as the total number of candidates must be greater than one for this calculation. For Challenge 7, the correct structure was one of three isomers and the ranking in first place was by chance. Four other challenges had only three isomers possible (Challenges 3, 4, 5 and 9) with the correct structure ranking second (Challenges 4 and 5) or third. The score, based on the fragmentation, log *K_ow_* and steric energy, was unable to provide sufficient decision-making strength to separate positional isomers of aromatic compounds. So far, no CASE via MS approach has matched the success of the established MS databases such as NIST, which are typically able to identify common positional isomers correctly. The results for Challenge 10 were similar to those above, with six possible isomers instead of three; the correct one was ranked in second place, also by chance.

The challenges with more structural candidates were more interesting and the additional parameters used in the scoring were valuable in improving the rank of the correct structures. Challenges 2, 8 and 15 had the correct structure in first place with over 10 candidates (80, 90 and 13, respectively) that were not just positional isomers. All three challenges were aromatic systems and as such, the steric energy component of the consensus score was the part that determined the correct rank. Challenge 11 also had the correct candidate in first place, but in this case the partitioning behaviour was the critical factor that separated correct from incorrect; only three of the 45 candidates were within the log*K_ow_* range given, and the correct candidate had the highest match value of the three. The RRP of these four challenges (2, 8, 11 and 15) is 0 and compares very favourably with the average of 0.273 for MOLGEN–MS. Challenge 6 had the correct candidate fifth of 187 and the RRP = 0.021 is also much lower (and thus better) than the MOLGEN–MS average RRP using fragmentation patterns alone. This was also an aromatic structure where the steric energy assisted in elevating the rank of the correct structure. However, in this case all candidates had very similar match values and there were six candidates with low steric energy. This resulted in the rank of 5*^th^*. Although Challenge 1 also had RRP below 0.273, the absolute rank (and thus RRP) could be improved using additional information from partitioning or retention behaviour; the former was not given and we did not incorporate the latter as this is not part of the MOLGEN products *per se*. Unlike in Challenge 2, the steric energy was not enough to separate the correct candidate from the incorrect structures here. Finally, the poor results for Challenge 13 (RRP = 1, *i.e*., ranked last) resulted from a quite simple but symmetrical structure with unspecific substructure classifiers. As a result of the symmetry, fewer fragments were predicted for this structure than for the other structural candidates. As the molecules were all similar, the steric energy and partitioning behaviour had no significant effect on the ranking and the match value determined the ranking. This effect was seen quite often in [32], but this has not yet been successfully incorporated into a modified match value.

## 4. Conclusions and Perspectives

Although the MOLGEN entries would not have won CASMI, even if considered as an official participant, these entries demonstrate that CASE via MS is certainly possible. The success of this approach depends strongly on the retrieval of very good substructure information from the spectrum. While this is automated for GC–MS with MOLGEN–MS, this is not yet the case for high accuracy MS/MS data, although very interesting developments have been made in recent years with interpretation of fragmentation trees (e.g., [35,36]) as well as maximum common substructures (e.g., [37,38]). The results of Category 2, performed by hand here and using experience rather than automated interpretation, provided perhaps a best case scenario for these challenges due to one of the authors’ prior experience with some of these compounds. Unlike some other structure generators that have been used recently for CASE via MS/MS (e.g., [38,39]), the fact that MOLGEN allows overlapping substructures and multiple good list (prescribed) and bad list (forbidden) entries (in MOLGEN terms) is a distinct advantage and is extremely valuable in structure elucidation via MS and MS/MS, where substructure information is often limited.

The maximum common substructure approach is an interesting alternative approach to the “hand-picked” substructures used here and is a good starting point to obtain clues for the identity of an unknown. However, it could be easy to neglect very similar substructures (e.g., positional isomers) that have the same or similar spectra or fragmentation patterns. These oversights could result in a significant underestimation of the number of candidates possible and lead to overly optimistic success rates that do not always represent the real situation accurately. Alternatively, a very small maximum common substructure could be the result, which would not provide sufficient restriction for structure generation. Although one could say for natural products that many of the common groups are known, in reality these are only the metabolites that have been identified so far and hundreds of thousands of metabolites remain unidentified [40]. In the majority of cases, MS/MS databases are still too small to determine conclusively whether positional isomers of a maximum common substructure would have the same fragmentation patterns or not. When using the maximum common substructure approach, caution is needed to “relax” the information in the maximum common substructure to accurately reflect the information one could expect from a mass spectrum.

All in all, the results shown here and in recent publications indicate that CASE via MS and especially HR–MS is certainly plausible and is an area that needs to be pursued actively in current and future research. It would be very interesting for future CASMIs if other research groups using structure generation approaches would participate to allow a real comparison of the results using different approaches and generators, rather than only speculating about advantages and disadvantages of different approaches.

## Figures and Tables

**Figure A2:**
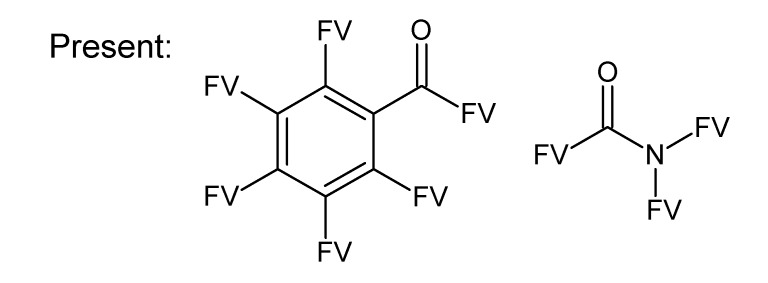
Substructures for GC–MS Challenge 2. FV = free valence.

**Figure A3:**
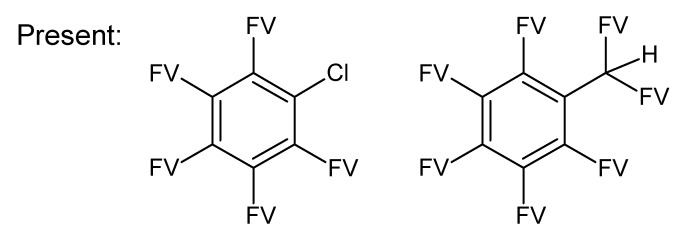
Substructures for GC–MS Challenges 3 and 4. FV = free valence.

**Figure A4:**
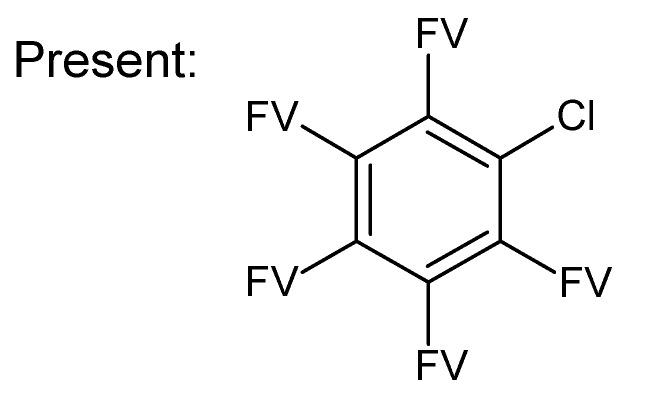
Substructures for GC–MS Challenge 5. FV = free valence.

**Figure A5:**
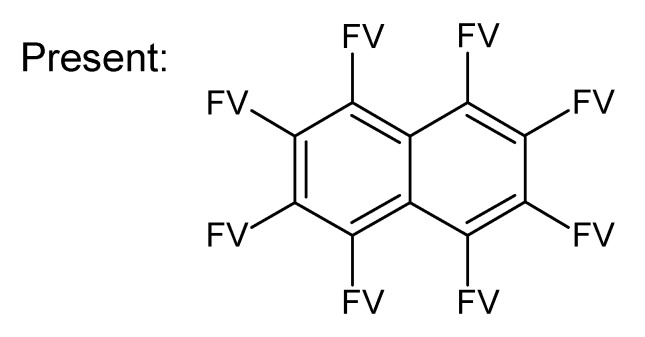
Substructures for GC–MS Challenge 6. FV = free valence.

**Figure A6:**
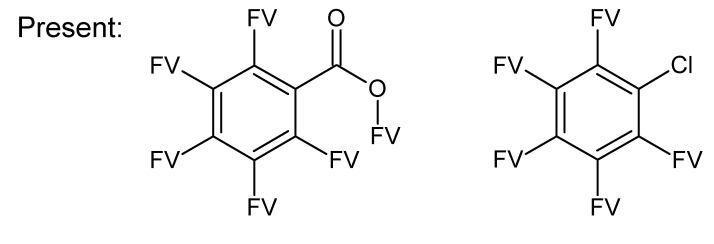
Substructures for GC–MS Challenge 7. FV = free valence.

**Figure A7:**
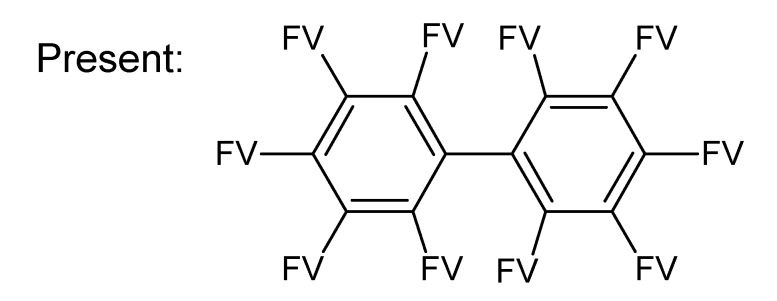
Substructures for GC–MS Challenge 8. FV = free valence.

**Figure A8:**
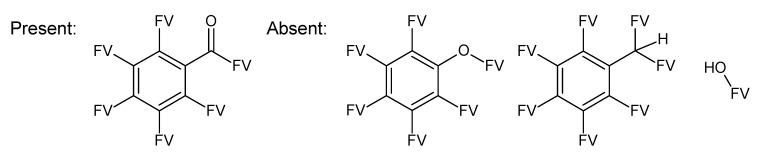
Substructures for GC–MS Challenge 9. FV = free valence.

**Figure A9:**
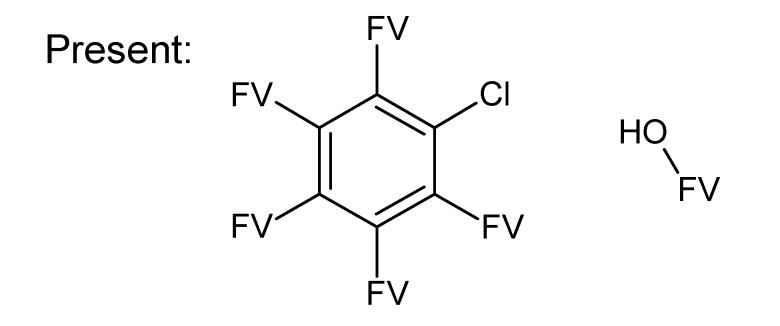
Substructures for GC–MS Challenge 10. FV = free valence.

**Figure A10:**
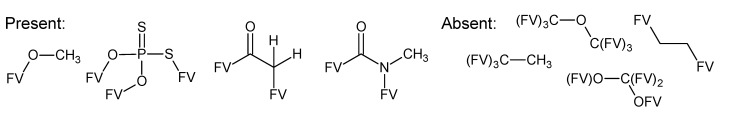
Substructures for GC–MS Challenge 11. FV = free valence.

**Figure A11:**
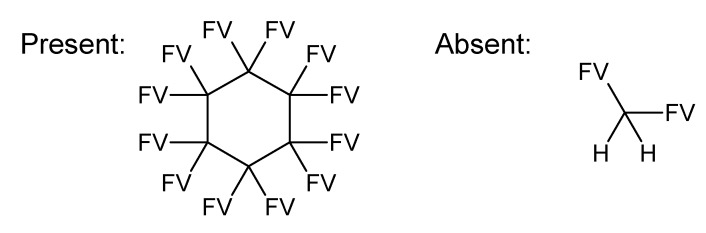
Substructures for GC–MS Challenge 12. FV = free valence.

**Figure A12:**
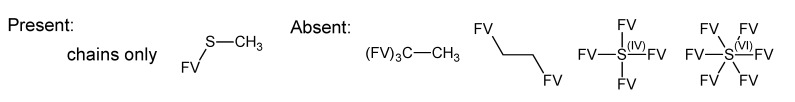
Substructures for GC–MS Challenge 13. FV = free valence.

**Figure A13:**
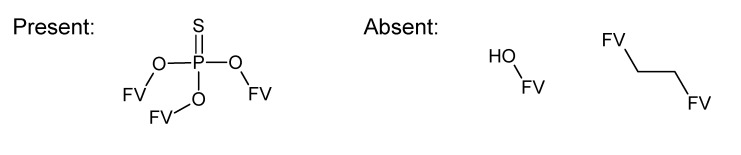
Substructures for GC–MS Challenge 14. FV = free valence.

**Figure A14:**
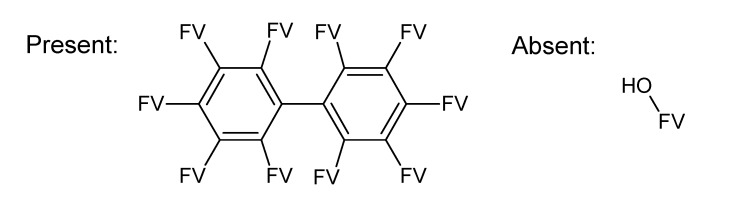
Substructures for GC–MS Challenge 15. FV = free valence.

## A. Appendix

### A.1. Substructures for CASMI Category 4: GC Challenges

The substructures used for preparing the entries for CASMI Category 4, structure identification using GC–MS, are given in Figures A1–A15.

## Supplementary Files

Supplementary File 1 Supporting Information: (PDF, 128 KB)
