# Supplementary material for: Small Molecule Identification with MOLGEN and Mass Spectrometry
Source: Metabolites. 2013 May 28;3(2):440–62. doi: 10.3390/metabo3020440 (PMC3901272; doi:10.3390/metabo3020440)
Supplement: Supplementary File 1 — Supporting Information: (PDF, 128 KB) [file metabolites-03-00440-s001.pdf]

Small Molecule Identification with MOLGEN and Mass Spectrometry

Markus Meringer[1] and Emma L. Schymanski[2]*

[1]DLR: German Aerospace Center, Earth Observation Center (EOC), Münchner Strasse 20, D-82234 Oberpfaffenhofen–Wessling, Germany
[2]Eawag: Swiss Federal Institute of Aquatic Science and Technology, Überlandstrasse 133, CH-8600 Dübendorf, Switzerland.
* Author to whom correspondence should be addressed; emma.schymanski@eawag.ch, Tel +41 58 765 5537, Fax +41 58 765 5210

Supporting Information

This file contains supporting information for the abovementioned article and includes:

Table SI-1: The LC Challenges for CASMI 2012. Pages 1-3.

Table SI-2: The GC Challenges for CASMI 2012. Page 4.

[1]: E. L. Schymanski and S. Neumann. CASMI: Challenges and Solutions, Metabolites, in review.

Table SI-1: The LC Challenges for CASMI 2012. Source: [1].

Challenge 1<br>Kanamycin A<br>$C_{18}H_{36}N_4O_{11}$<br>PubChem: 6032<br>ChemSpider: 5810	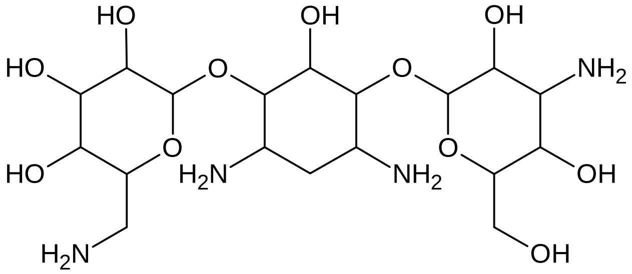
Challenge 2<br>1,2-Bis-O-sinapoyl-beta-D-glucoside<br>$C_{28}H_{32}O_{14}$<br>PubChem: 5280665<br>ChemSpider: 4444262	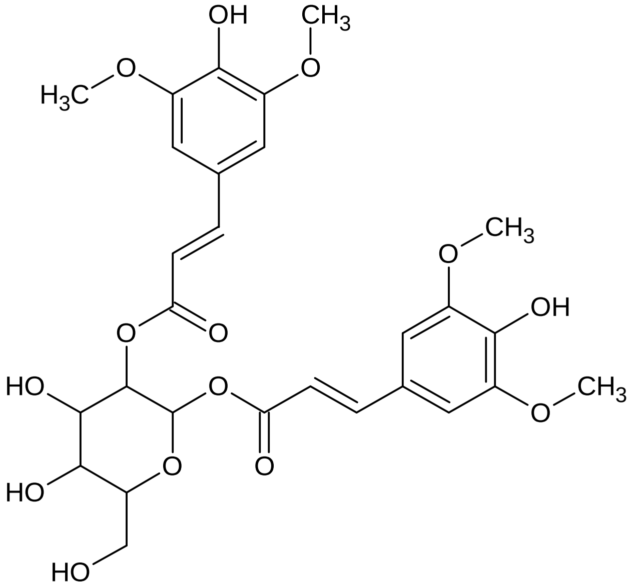

Challenge 3
Glucolesquerellin
$C_{14}H_{27}NO_9S_3$
PubChem: 46173875
ChemSpider: NA

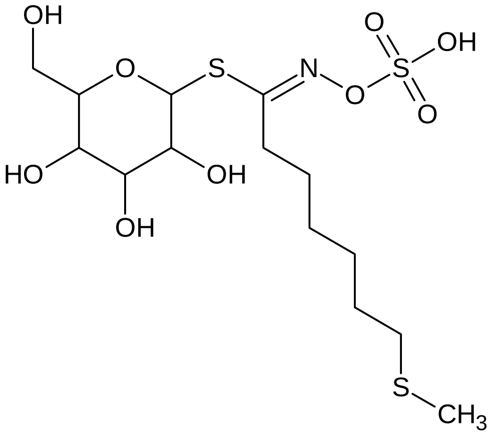

Challenge 4
Escholtzine
$C_{19}H_{17}NO_4$
PubChem: 12304178
ChemSpider: 16740500

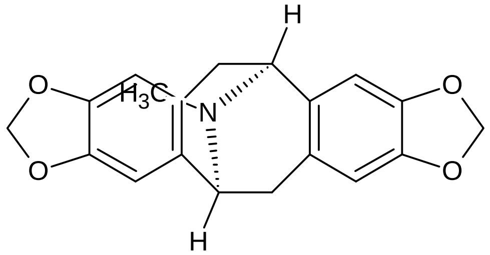

Challenge 5
Reticuline
$C_{19}H_{23}NO_4$
PubChem: 10233
ChemSpider: 9816

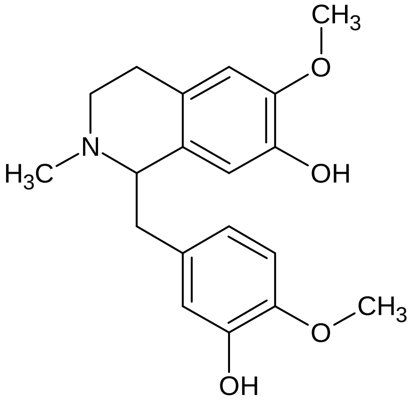

Challenge 6
Rheadine
$C_{21}H_{21}NO_6$
PubChem: 197775
ChemSpider: 171184

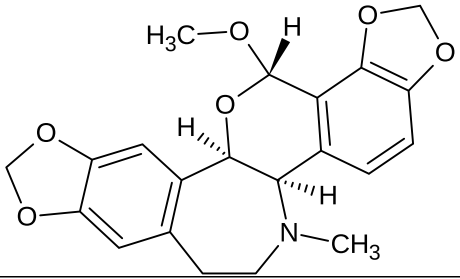

Challenge 10
1-Aminoanthraquinone
$C_{14}H_9NO_2$
PubChem: 6710
ChemSpider: 6454

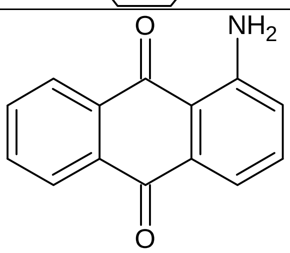

Challenge 11
1-Pyrenemethanol
$C_{17}H_{12}O$
PubChem: 104977
ChemSpider: 94729

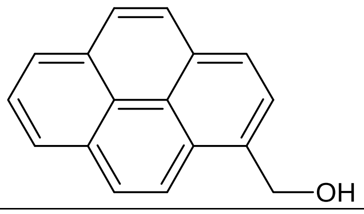

Challenge 12
alpha-(o-Nitro-p-tolyl azo)acetoacetanilide
$C_{17}H_{16}N_4O_4$
PubChem: 221491
ChemSpider: 192174

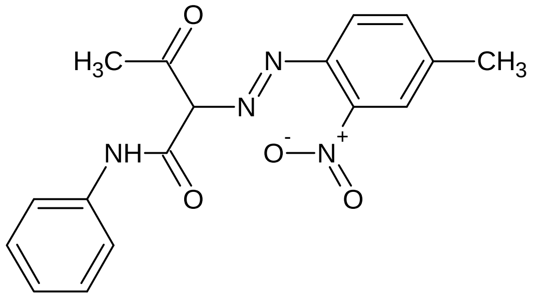

Challenge 13
Benzyl-diphenyl phosphine oxide
$C_{19}H_{17}OP$
PubChem: 76293
ChemSpider: 68772

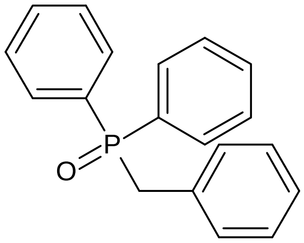

Challenge 14
1H-Benz[g]indole
$C_{12}H_9N$
PubChem: 98617
ChemSpider: 89061

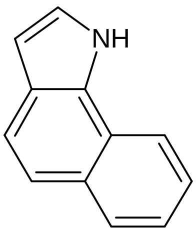

Challenge 15
1-Isopropyl-5-methyl-1H-indole-2,3-dione
$C_{12}H_{13}NO_2$
PubChem: 2145522
ChemSpider: 1606080

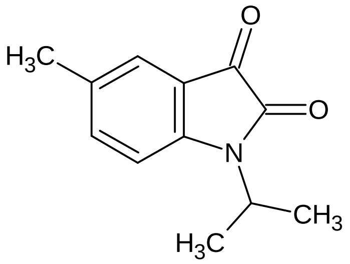

Challenge 16
1-[(4-Methoxyphenyl) amino]-1-oxo-2-propanyl 6-oxo-1-propyl-1,6-dihydro-3-pyridazinecarboxylate
$C_{18}H_{21}N_3O_5$
PubChem: 18091616
ChemSpider: 16896706

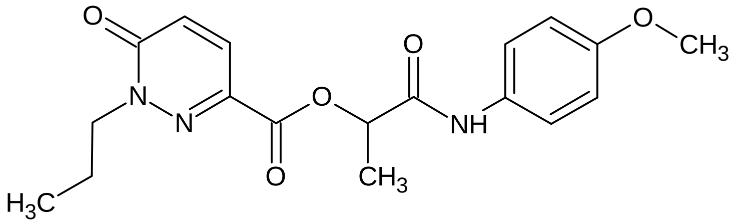

Challenge 17
Nitrin
$C_{13}H_{13}N_3$
PubChem: 68380
ChemSpider: 61666

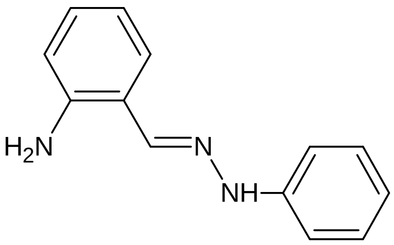

Table SI-2: The GC Challenges for CASMI 2012. Source: [23].

Challenge 1<br>Phthalic anhydride<br>$C_8H_4O_3$<br>PubChem: 6811<br>ChemSpider: 6552	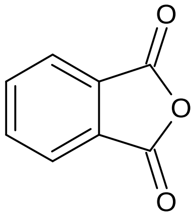	Challenge 2<br>Phthalimide<br>$C_8H_5NO_2$<br>PubChem: 6809<br>ChemSpider: 6550	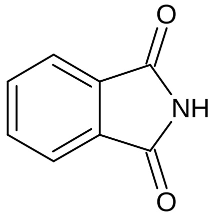
Challenge 3<br>2-Chlorobenzyl alcohol<br>C_7H_7ClO<br>PubChem: 28810<br>ChemSpider: 26799	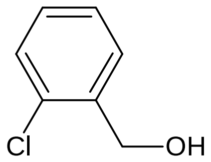	Challenge 4<br>4-Chlorobenzyl alcohol<br>C_7H_7ClO<br>PubChem: 13397<br>ChemSpider: 12823	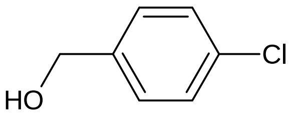
Challenge 5<br>1,4-Dichlorobenzene<br>$C_6H_4Cl_2$<br>PubChem: 4685<br>ChemSpider: 13866817	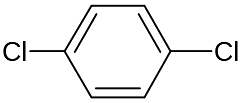	Challenge 6<br>Acenaphthene<br>$C_{12}H_{10}$<br>PubChem: 6734<br>ChemSpider: 6478	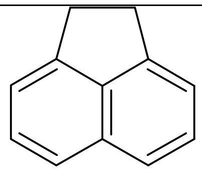
Challenge 7<br>4-Chlorobenzoic acid<br>$C_7H_5ClO_2$<br>PubChem: 6318<br>ChemSpider: 6079	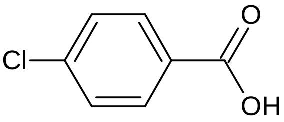	Challenge 8<br>Fluorene<br>$C_{13}H_{10}$<br>PubChem: 6853<br>ChemSpider: 6592	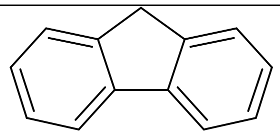
Challenge 9<br>Methyl 2-chlorobenzoate<br>$C_8H_7ClO_2$<br>PubChem: 11895<br>ChemSpider: 11402	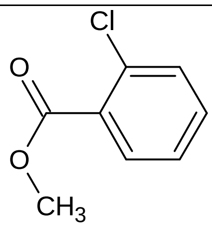	Challenge 10<br>2,4,6-Trichlorophenol<br>$C_6H_3Cl_3O$<br>PubChem: 6914<br>ChemSpider: 21106172	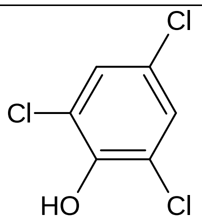
Challenge 11<br>Formothion<br>$C_6H_{12}NO_4PS_2$<br>PubChem: 17345<br>ChemSpider: 16412	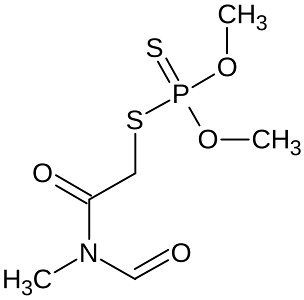	Challenge 12<br>alpha-Hexachloro-cyclohexane<br>$C_6H_6Cl_6$<br>PubChem: 727<br>ChemSpider: 10468511	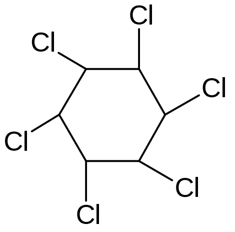
Challenge 13<br>Dimethyl carbonotrithioate<br>$C_3H_6S_3$<br>PubChem: 16840<br>ChemSpider: 15959	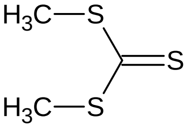	Challenge 14<br>O,O,O-Trimethyl thiophosphate<br>$C_3H_9O_3PS$<br>PubChem: 9038<br>ChemSpider: 8686	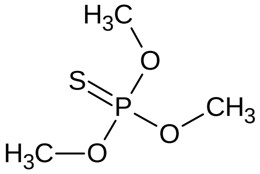
Challenge 15<br>Dibenzofuran<br>$C_{12}H_8O$<br>PubChem: 568<br>ChemSpider: 551	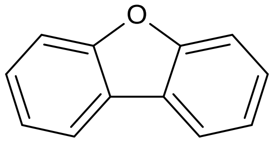	Challenge 16<br>O,S,S-Trimethyl phosphorodithioate<br>$C_3H_9PS_2O_2$<br>PubChem: 31435<br>ChemSpider: 29165	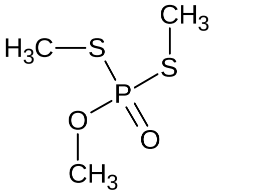
